# Investigation of the pharmacological effect and mechanism of mountain-cultivated ginseng and garden ginseng in cardiovascular diseases based on network pharmacology and zebrafish experiments

**DOI:** 10.3389/fphar.2022.920979

**Published:** 2022-09-01

**Authors:** Ting Yu, Yan-Xin Zhang, Xin-Juan Liu, Dan-Qing Chen, Dan-Dan Wang, Guo-Qin Zhu, Qi Gao

**Affiliations:** ^1^ Shanghai University of Traditional Chinese Medicine, Shanghai, China; ^2^ SPH XingLing Sci&Tech. Pharmaceutical Co., Ltd., Shanghai, China; ^3^ Shanghai SPH Shenxiang Health Medicine Co., Ltd., Shanghai, China

**Keywords:** MCG, GG, UPLC-Q-TOF/MS, network pharmacology, zebrafish, CVD

## Abstract

Ginseng (*Panax ginseng* C.A. Mey) is a kind of perennial herb of the *Panax* genus in the Araliaceae family. The secondary metabolites of mountain-cultivated ginseng (MCG) and garden ginseng (GG) vary greatly due to their different growth environments. To date, the differences in their pharmacological effects on cardiovascular diseases (CVDs) and their clinical applications remain unclear. To distinguish between the components of MCG and GG, ultra-high-performance liquid chromatography-quadrupole time-of-flight tandem mass spectrometry (UPLC-Q-TOF/MS) was performed. Next, the relationship between the expression of metabolites and the categories of the sample were analyzed using supervised partial least squares discriminant analysis and orthogonal partial least squares discriminant analysis. A network-based pharmacology approach was developed and applied to determine the underlying mechanism of different metabolites in CVD. In the present study, the role of MCG and GG in angiogenesis and their protective effects on damaged blood vessels in a vascular injury model of zebrafish were investigated. Using UPLC-Q-TOF/MS, 11 different metabolites between MCG and GG were identified. In addition, 149 common target genes associated with the metabolites and CVD were obtained; these targets were related to tumor protein P53, proto-oncogene tyrosine-protein kinase Src, human ubiquitin-52 amino acid fusion protein, ubiquitin-40S ribosomal protein S27a, polyubiquitin B, signal transducer and activator of transcription 3, isocitrate dehydrogenase 1, vascular endothelial growth factor A, glycose synthase kinase-3B, and coagulation factor II and were associated with the regulation of the phosphoinositide 3-kinase-Akt signaling pathway, the tumor necrosis factor signaling pathway, and the hypoxia-inducible factor-1 (HIF-1) signaling pathway, which play important roles in the curative effect in CVD treatment. Both types of ginseng can promote the growth of the subintestinal vessel plexus and protect injured intersegmental vessels through the HIF-1α/vascular endothelial growth factor signaling pathway in a dose-dependent manner. In addition, MCG has a stronger impact than GG. This is the first time metabolomics and network pharmacology methods were combined to study the difference between MCG and GG on CVDs, which provides a significant theoretical basis for the clinical treatment of CVD with two kinds of ginseng.

## 1 Introduction

As a traditional herbal medicine, ginseng (*Panax ginseng* C.A. Mey) has been used for the treatment of various ailments for the past 2000 years and is considered the “king of herbs” (Chopra et al., 2021). Based on the growth environment and cultivation methods, ginseng has been divided into three types: mountain wild ginseng, mountain-cultivated ginseng (MCG), and garden ginseng (GG) (Xu et al., 2016; Kim et al., 2018; Li et al., 2021). MCG is grown in natural environments, especially in high-altitude mountains, before being used as a medicine (Tu et al., 2017). GG is the ginseng cultivated under artificial planting conditions (Zhu et al., 2021). In addition, most MCG is collected for at least 15 years or longer, whereas GG is harvested for only 4–5 years. The different growth environments and the different types and contents of secondary metabolites lead to differences in the pharmacodynamic effect between the two kinds of ginseng (Guerriero et al., 2018; Kim et al., 2018; Fan et al., 2019). MCG and GG are widely available in the market and are used in clinics. Due to the scarcity of MCG, we investigated the necessity of the precise clinical application of ginseng in the treatment of cardiovascular diseases (CVDs) according to the perspective of the clinical application of ginseng.

CVDs (Teo and Dokainish, 2017) are the leading cause of death worldwide. Atherosclerosis, acute myocardial infarction, hypertension, myocardial ischemia, and other CVDs (Li et al., 2018) affect around 795,000 people in the United States each year. Because of its antiatherosclerotic, antiarrhythmic, and anti-myocardial ischemia actions as well as its inhibitory effect on ventricular remodeling, ginseng (Irfan et al., 2020; Shaito et al., 2020; Zhu et al., 2021) may also be used in the treatment of CVDs. Through the building of a multilevel network of “drug target path,” network pharmacology (Hopkins, 2008; Li et al., 2018; WU et al., 2022) can be used to analyze pharmacologically active ingredients, compounds, and possible molecular network mechanisms in traditional Chinese medicine. Due to the high degree of genetic, physiological, and pharmacological similarity to humans, zebrafish are commonly and widely used as a typical animal disease model and have been involved in several cases of drug discovery; thus, zebrafish are frequently used in the investigation of disease treatment and drug discovery (MacRae and Peterson, 2015; Zhang et al., 2019). In this study, using network pharmacology and a zebrafish disease model, we investigated the possible molecular mechanism of MCG and GG in the treatment of CVD and provided an important basis for the clinical application of ginseng in the treatment of CVD.

## 2 Materials and methods

### 2.1 Analysis of ultra-high-performance liquid chromatography-quadrupole time-of-flight tandem mass spectrometry

The samples of MCG (*n* = 11, 20 years) and GG (*n* = 11, 4 years) were obtained from Huanren Liaoning, China. All samples were provided by Shanghai SPH Shen Xiang Health Medicine Co., Ltd. and were identified by Professor Yuexiong Li. After the addition of 50 mg of MCG and GG powder to 75% cold methanol solution and crushing at low temperature with a high flux crushing instrument, the mixtures were extracted using ultrasonics for 30 min at 4°C. The solution was then centrifuged at 13,000 × g for 15 min at 4°C to obtain the supernatant. At last, the supernatant was filtered using a 0.22-μm filter membrane and injected directly into the ultra-high-performance liquid chromatography-quadrupole time-of-flight tandem mass spectrometry (UPLC-Q-TOF/MS) system for analysis.

Chromatographic separation was performed using an Acquity BEH C18 column (100 mm × 2.1 mm id, 1.7 μm; Waters, Milford, MA, United States). Separation was achieved using the following gradient: 5% B—25% B—25% B—100% B—1.5–10.0 min, 100% B—100% B—10.0–13.0 min; 100% B—5% B—13.0–13.5 min and 13.5–14.5 min, holding at 5% B at a flow rate of 0.40 ml/min, where B was acetonitrile (0.1% (v/v) formic acid) and A was aqueous formic acid (0.1% (v/v) formic acid) in the positive ion mode and B was acetonitrile (containing 5 mM ammonium formate) and A was water (containing 5 mM ammonium formate) in the negative ion mode. The injection volume was 3 μL, and the column temperature was set at 45.0°C. The mass spectrometric data were collected using an LTQ Orbitrap mass spectrometer equipped with an electrospray ionization source operating in either positive or negative ion mode. The capillary and source temperatures were set at 350°C, with a desolvation gas flow of 45 L/h. Centroid data were collected from 50 to 1,000 m/z with a resolution of 30,000. The data were analyzed using QI software.

### 2.2 Network pharmacology analysis

#### 2.2.1 Target identification and network construction

The different ingredients between MCG and GG were imported into the Traditional Chinese Medicine Systems Pharmacology database (http://lsp.nwu.edu.cn/tcmsp.php), SymMap database (http://www.symmap.org/), Encyclopedia of Traditional Chinese Medicine database (http://www.nrc.ac.cn:9090/ETCM/), and TCM-MESH database (http://mesh.tcm.microbioinformatics.org/). For the components that were not included in these databases, the Swiss Target Prediction (http://swisstargetprediction.ch/) and STITCH (http://stitch.embl.de/) databases were applied to predict the targets according to the chemical similarity. DrugBank (https://www.drugbank.ca/) and HGDB (http://www.hgdb.ir/Default1.aspx) databases were used to collect CVD targets. A Venn diagram of the overlapping relationship among the targets, the “components-targets” network, and the protein–protein interaction (PPI) network was established.

#### 2.2.2 Kyoto encyclopedia of genes and genomes enrichment analysis and molecular docking

The key proteins were placed in the database for annotation, visualization and integrated discovery (DAVID) 6.8 for Kyoto Encyclopedia of Genes and Genomes (KEGG) enrichment analysis. The KEGG database could identify functions at a higher level and the biological information resources that interact with them at the genomic and molecular levels. The results of the enrichment analysis of the study were selected at *p* < 0.05 and excluded specific disease pathways. All the structures of the compounds were downloaded from the PubChem database, and protein crystal structures were obtained from the protein data bank database. Based on the binding scores, good binding activity (binding score < 7) and strong binding activity (binding score < 9) were defined.

### 2.3 Experiment verification

#### 2.3.1 Reagents and materials

The vatalanib dihydrochloride PTK787 was obtained from Selleck Chemicals (Shanghai, China). Methyl sulfoxide, HCl, glycerin, sodium dodecyl sulfate, NaCl, KCl, Na_2_HPO_4_·12H_2_O, and KH_2_PO_4_ were purchased from Sinopharm (Shanghai, China). XueSaiTong (XST) was obtained from TeAnNa (Yunnan, China). Trizol was obtained from Invitrogen (Wuhan, China). Analytical-grade methanol, acetonitrile, and formic acid were purchased from Merck (Darmstadt, Germany).

#### 2.3.2 Animals

The transgenic zebrafish line Tg (fli1a: EGFP) y1 was obtained from China Zebrafish Resource Center (Wuhan, China), and enhanced green fluorescent protein–expressed endothelial cells were used for angiogenesis observations. The zebrafish were maintained with a 14/10 h light–dark cycle and were fed brine shrimp twice a day. The zebrafish embryos were produced by natural pairing and were incubated in an embryo culture medium at 28.5°C.

#### 2.3.3 Proangiogenic effect of mountain-cultivated ginsengand garden ginseng in zebrafish

Healthy embryos that developed 24 h after fertilization (hpf) were collected and treated with various concentrations of MCG and GG (25, 50, and 100 μg/ml) for 48 h, and the number of sprouted and crossed vessels of the subintestinal vessel plexus (SIV) was counted in each zebrafish embryo. A stereoscopic fluorescence microscope (Nikon, Shanghai, China) was used for observation of the growth of SIVs. The embryos at 21 hpf were pretreated with 0.2 μg/ml PTK787 (VEGF receptor inhibitor) for 3 h and then treated with various concentrations of MCG and GG (25, 50, and 100 μg/ml) for 48 h. The elongation of intersegmental vessels (ISVs) from the dorsal aorta to the dorsal longitudinal anastomosis was observed, and the length of ISVs of each embryo was measured. XST, which mainly comprised saponins extracted from *Panax notoginseng* [(Burkill) F. H. Chen] was used as the positive control group. At least six embryos were analyzed in each group, and the experiments were replicated three times.

#### 2.3.4 Real-time RT-PCR

Each zebrafish embryo sample was collected in three replicates with 50 embryos per group. Total RNA was extracted using the Trizol extraction kit, and cDNA was synthesized from total RNA using the EVO M-MLV RT kit (Shanghai, China). The cDNA was diluted 10 times before RT-PCR analysis. Quantitative analysis of the mRNA of vascular endothelial growth factor receptor 2 (VEGFR2), hypoxia-inducible factor-1α (HIF-1α), interleukin-6 (IL-6), interleukin-1β (IL-1β), tumor necrosis factor-α (TNF-α), and glyceraldehyde-3-phosphate dehydrogenase (GAPDH) was performed using the Light Cycle 96 Real-Time PCR platform, and each gene was normalized to the internal control gene GAPDH. The results were analyzed using the 2^−ΔΔCt^ relative quantification method. The primer sequences that were used are presented in Table 3.

#### 2.3.5 Statistical analysis

All data were analyzed using GraphPad Prism 8.0 and are presented as the mean ± standard error of the mean. Two-sided unpaired Student’s t-test and one-way analysis of variance (ANOVA), followed by the Holm–Sidak test, were used to determine the difference between two independent samples and more than two independent samples, respectively. Comparisons between groups were analyzed using a two-way ANOVA, followed by Bonferroni’s post-hoc multiple comparison test. The correlations were calculated using Pearson correlation analysis. *p* < 0.05 was considered statistically significant.

## 3 Results

### 3.1 Identification of the different metabolites between mountain-cultivated ginseng and garden ginseng

**FIGURE 1:**
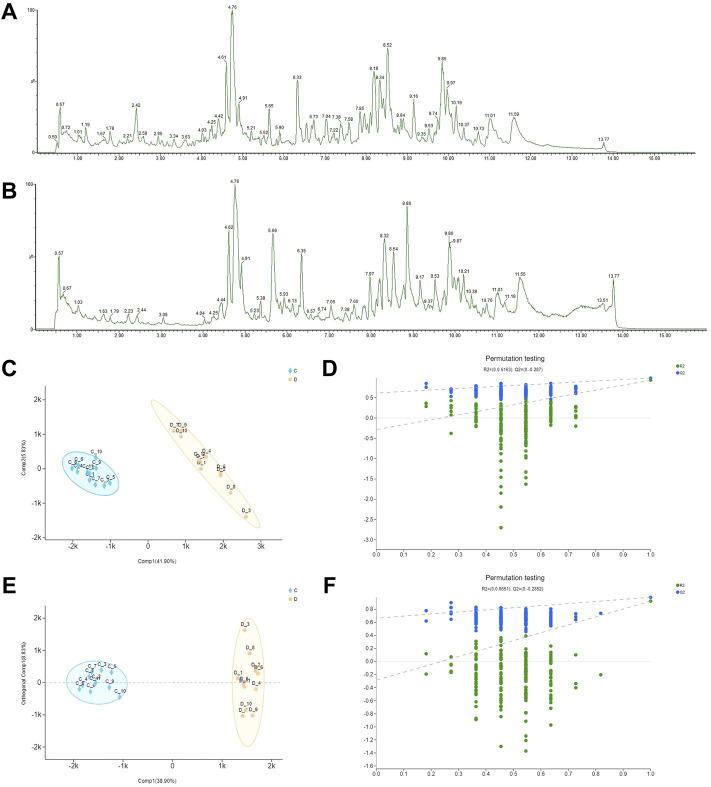
Differential metabolites between mountain-cultivated ginseng (MCG) and garden ginseng (GG) determined by ultra-high-performance liquid chromatography-quadrupole time-of-flight tandem mass spectrometry. The total ion chromatograms of MCG and GG in negative ions. **(A)** MCG and **(B)** GG. The results of statistical analysis from the ginseng samples. **(C)** Partial least squares discriminant analysis (PLS-DA) score plot, **(D)** permutation test, **(E)** orthogonal partial least squares discriminant analysis (OPLS-DA) score plot, and **(F)** permutation test.

The total ion chromatograms of MCG and GG in negative ions are presented in Figures 1A,B. All metabolite components were matched in the QI software, as shown in Supplementary Table S1, and 91 total metabolite components were obtained. To analyze the relationship between the metabolite level and sample categories, supervised partial least squares discriminant analysis (PLS-DA) was employed, and the metabolite profiles of MCG and GG were separated. Q^2^ > 0.5 proves that the model is reasonable and effective, and can be used for data analysis (R^2^X = 0.477, R^2^Y = 0.985, Q^2^ = 0.931) (Figures 1C, D). Orthogonal partial least squares discriminant analysis (OPLS-DA) was also used to explore candidate biomarkers related to ginseng by comparing two groups. The MCG and GG samples were clearly divided into two groups. Cross-validation of model showed that Q^2^ > 0.5, the model is reasonable and effective, and can be used for data analysis (R^2^X = 0.477, R^2^Y = 0.921, Q^2^ = 0.927) (Figures 1E, F). The importance of the variable in the projection (VIP) was introduced according to the OPLS-DA model to further study the classification contribution variable index and detect differential metabolites. VIP >1 indicated a significant difference, and a *t*-test was performed on different screened metabolites. When *p* < 0.05, it indicated a significant difference and was considered statistically significant. Both PLS-DA and OPLS-DA were conducted by SIMCA 14.1 software (Umetrics, China). Based on the above analysis (VIP >1, *p* < 0.05), 11 different metabolites were analyzed (Table 1).

**TABLE 1:** Eleven differential metabolites between mountain-cultivated ginseng and garden ginseng.

No.	t_R_ (min)	Precursor ion and/or adduct ions	Error (ppm)	Formula	Identification	VIP value
1	8.83	409.2293 [M-H]^-^	−16.45	C_19_H_39_O_7_P	1-Palmitoyl Lysophosphatidic Acid	6.3956
2	7.08	829.4938 [M -H + HCOOH]^-^	−4.39	C_42_H_72_O_13_	Ginsenoside Rg3	5.3053
3	3.89	991.5495 [M -H + HCOOH]^-^	2.88	C_48_H_82_O_18_	Ginsenoside Re	4.2148
4	5.82	991.5476 [M -H + HCOOH]^-^	1.00	C_48_H_82_O_18_	Ginsenoside B2	3.9941
5	5.07	815.4793 [M -H + HCOOH]^-^	−0.97	C_41_H_70_O_13_	Ginsenoside F5	3.0355
6	6.84	829.4942 [M -H + HCOOH]^-^	−0.97	C_42_H_72_O_13_	Ginsenoside C	2.1929
7	0.76	191.0189 [M-H]^-^	−4.54	C_6_H_8_O_7_	Citric acid	1.9622
8	0.59	173.0921 [M-H]^-^	−5.88	C_7_H_14_N_2_O_3_	N2-Acetyl-L-ornithine	1.3699
9	8.87	783.3188 [M-H-2Glc-Xyl]-	5.74	C_59_H_100_O_27_	Notoginsenoside Fa	1.3030
10	1.58	164.0718 [M-H]^-^	0.41	C_9_H_11_NO_2_	L-Phenylalanine	1.1431
11	8.71	689.4093 [M + Cl]^-^	8.50	C_36_H_62_O_10_	Ginsenoside M7cd	1.0106

### 3.2 Network pharmacology analysis

#### 3.2.1 Result of network construction

**FIGURE 2:**
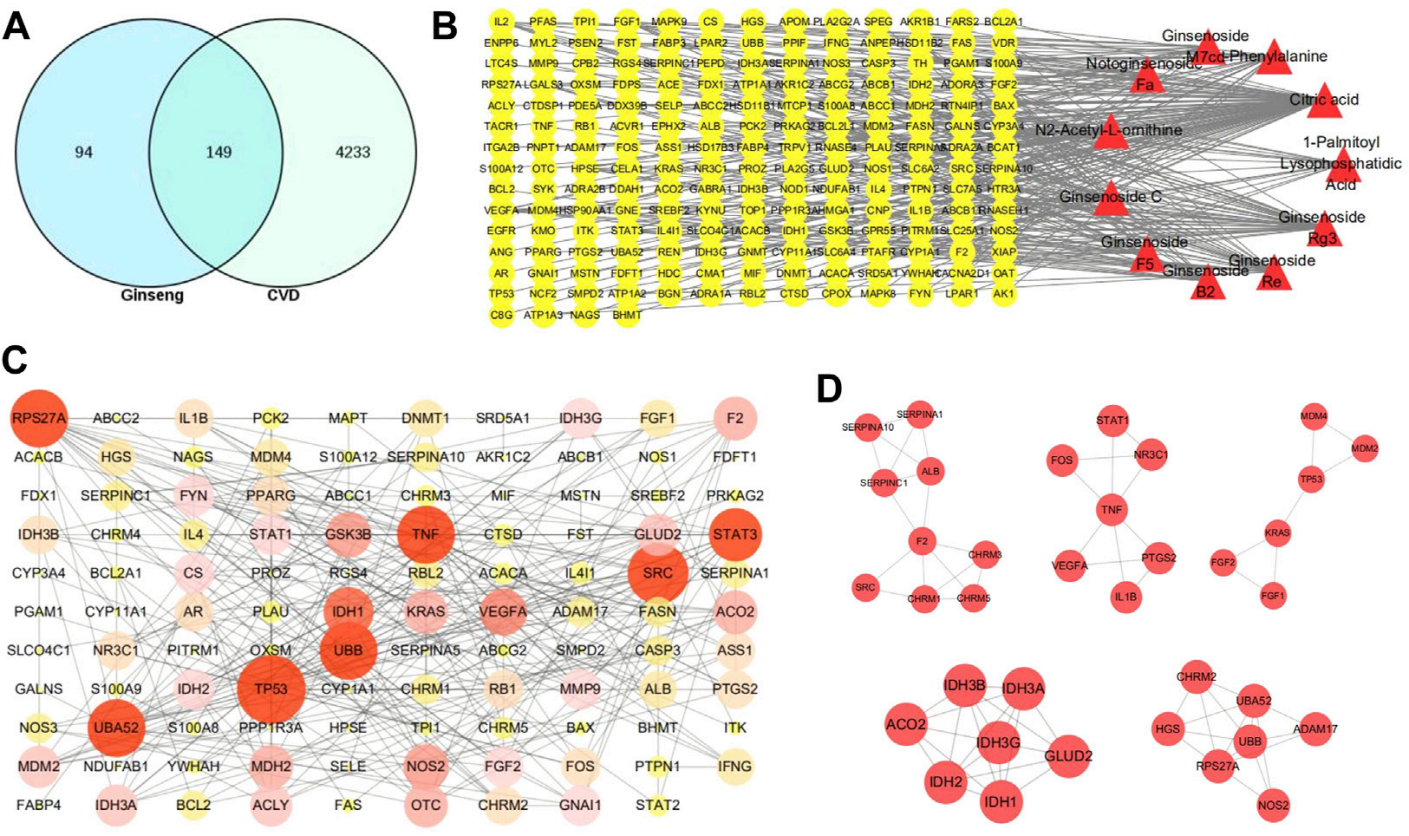
Target screening and network construction. **(A)** The venn diagram of the targets of gingseng and cardiovascular diseases (CVDs). **(B)** “Components-targets” network. The yellow circles represent the common targets of gingseng and CVD; the red triangle represents the differential components between MCG and GG. **(C)** Protein–protein interaction (PPI) network. The higher the degree value, the more red the color and the larger the shape. **(D)** Five closely connected submodules in the network. The 5-nucleon module is the interaction between tumor protein receptors, the 3-nucleon and 1-nucleon module is the interaction between cardiovascular pathway-related proteins, and the 2-nucleon module is the interaction between inflammation-related proteins.

A total of 243 targets for different compounds were obtained; the DrugBank and HGDB databases were used to identify 4382 CVD-related targets, and the UniProt database was used to collect the gene names of all targets. In addition, the null and repetitive targets were removed. All compound targets were assigned to CVD target genes to obtain 149 common target genes (Figure 2A; Supplementary Table S3).

The “Components-targets” network was then constructed to determine the interaction between components and targets. This network contained 197 nodes and 291 edges; the more edges a component or target had, the more important they were in this network. The network showed that citric acid, ginsenoside Rg3, and ginsenoside Re might be the most important components between MCG and GG in CVD (Figure 2B). The topological properties of different components are shown in Supplementary Table S2. Based on the results of STRING for the interaction of targets, we built the PPI network (Figure 2C). The PPI network was clustered by cluster analysis using the MCOED plug-in, and five clusters were used to analyze the CVD mechanism. The top 10 targets were tumor protein P53 (TP53), proto-oncogene tyrosine-protein kinase Src, human ubiquitin-52 amino acid fusion protein, ubiquitin-40S ribosomal protein S27a, polyubiquitin B, signal transducer and activator of transcription 3 (STAT3), isocitrate dehydrogenase 1, vascular endothelial growth factor A (VEGFA), glycose synthase kinase-3B, and coagulation factor II (Figure 2D).

#### 3.2.2 Kyoto encyclopedia of genes and genomes enrichment analysis and molecular docking

KEGG enrichment analysis results are shown in Table 2; 59 pathways were obtained after removing the results of specific disease pathways. The top 15 KEGG analysis results were identified with *p* < 0.05, which served as a threshold, including the phosphoinositide 3-kinase (PI3K)-Akt signaling pathway, TNF signaling pathway, insulin resistance mechanism, and HIF-1 signaling pathway. The pathway with the most enriched genes was the PI3K-Akt signaling pathway, which interacted with 15 genes; VEGFA is an important protein factor in this signaling pathway.

**TABLE 2:** Top 15 pathways for Kyoto Encyclopedia of Genes and Genomes analysis.

Term	Count	*p*-value	Genes
Metabolic pathways	34	9.43E-04	OTC, BCAT1, HSD17B3, GLUD2, EPHX2, ACACA, OAT, NOS2, PLA2G5, CYP11A1, SMPD2, FDFT1, TH, AK1, NOS1, NOS3, LTC4S, NDUFAB1, CYP3A4, KYNU, GNE, HSD11B2, ANPEP, OXSM, ASS1, HSD11B1, PFAS, ACACB, HPSE, NAGS, FDPS, PLA2G2A, PTGS2, KMO
Pathways in cancer	26	1.37E-07	RB1, MAPK8, FOS, BCL2, BCL2L1, MMP9, F2, NOS2, LPAR2, XIAP, HSP90AA1, IFNG, FGF1, BAX, IL4, FGF2, IL2, VEGFA, LPAR1, CASP3, MAPK9, EGFR, STAT3, PPARG, ITGA2B, PTGS2
PI3K-Akt signaling pathway	15	1.42E-03	BCL2, BCL2L1, SYK, LPAR2, HSP90AA1, FGF1, NOS3, IL4, FGF2, IL2, VEGFA, LPAR1, RBL2, EGFR, ITGA2B
Fluid shear stress and atherosclerosis	13	7.77E-07	MAPK8, FOS, BCL2, MMP9, HSP90AA1, IL1B, IFNG, NOS3, TNF, VEGFA, ASS1, MAPK9, ITGA2B
IL-17 signaling pathway	11	9.20E-07	MAPK8, FOS, MMP9, HSP90AA1, IL1B, IFNG, IL4, TNF, CASP3, MAPK9, PTGS2
Necroptosis	11	7.89E-05	MAPK8, BCL2, GLUD2, XIAP, HSP90AA1, IL1B, IFNG, BAX, TNF, MAPK9, STAT3
MAPK signaling pathway	11	4.42E-03	MAPK8, FOS, CACNA2D1, IL1B, FGF1, FGF2, TNF, VEGFA, CASP3, MAPK9, EGFR
AGE-RAGE signaling pathway in diabetic complications	10	1.28E-05	MAPK8, BCL2, IL1B, NOS3, BAX, TNF, VEGFA, CASP3, MAPK9, STAT3
Sphingolipid signaling pathway	10	5.18E-05	MAPK8, BCL2, SMPD2, ADORA3, FYN, NOS3, BAX, TNF, ABCC1, MAPK9
Small cell lung cancer	9	4.36E-05	RB1, BCL2, BCL2L1, NOS2, XIAP, BAX, CASP3, ITGA2B, PTGS2
Th17 cell differentiation	9	1.09E-04	MAPK8, FOS, HSP90AA1, IL1B, IFNG, IL4, IL2, MAPK9, STAT3
Apoptosis	9	4.47E-04	MAPK8, FOS, BCL2, BCL2L1, XIAP, BAX, TNF, CASP3, MAPK9
T-cell receptor signaling pathway	8	5.69E-04	MAPK8, FOS, FYN, IFNG, IL4, IL2, TNF, MAPK9
TNF signaling pathway	8	5.20E-04	MAPK8, FOS, MMP9, IL1B, TNF, CASP3, MAPK9, PTGS2
HIF-1 signaling pathway	7	3.10E-03	BCL2, NOS2, IFNG, NOS3, VEGFA, EGFR, STAT3

VEGFA is a homologous dimer vasoactive glycoprotein and a key mediator of angiogenesis and vessel permeability (Braile et al., 2020). Research has found that VEGFA can improve microvascularization and oxygen diffusion to limit the adverse consequences of cardiac ischemia (Żak et al., 2019). In addition, VEGFA has been clinically used as a potential circulating biomarker for CVD (Kikuchi et al., 2019). Our results demonstrated that ginsenosides Re, B2, and C could protect against CVD through the PI3K-Akt signaling pathway, TNF signaling pathway, insulin resistance mechanism, and NF-kappa B signaling pathway.

**FIGURE 3:**
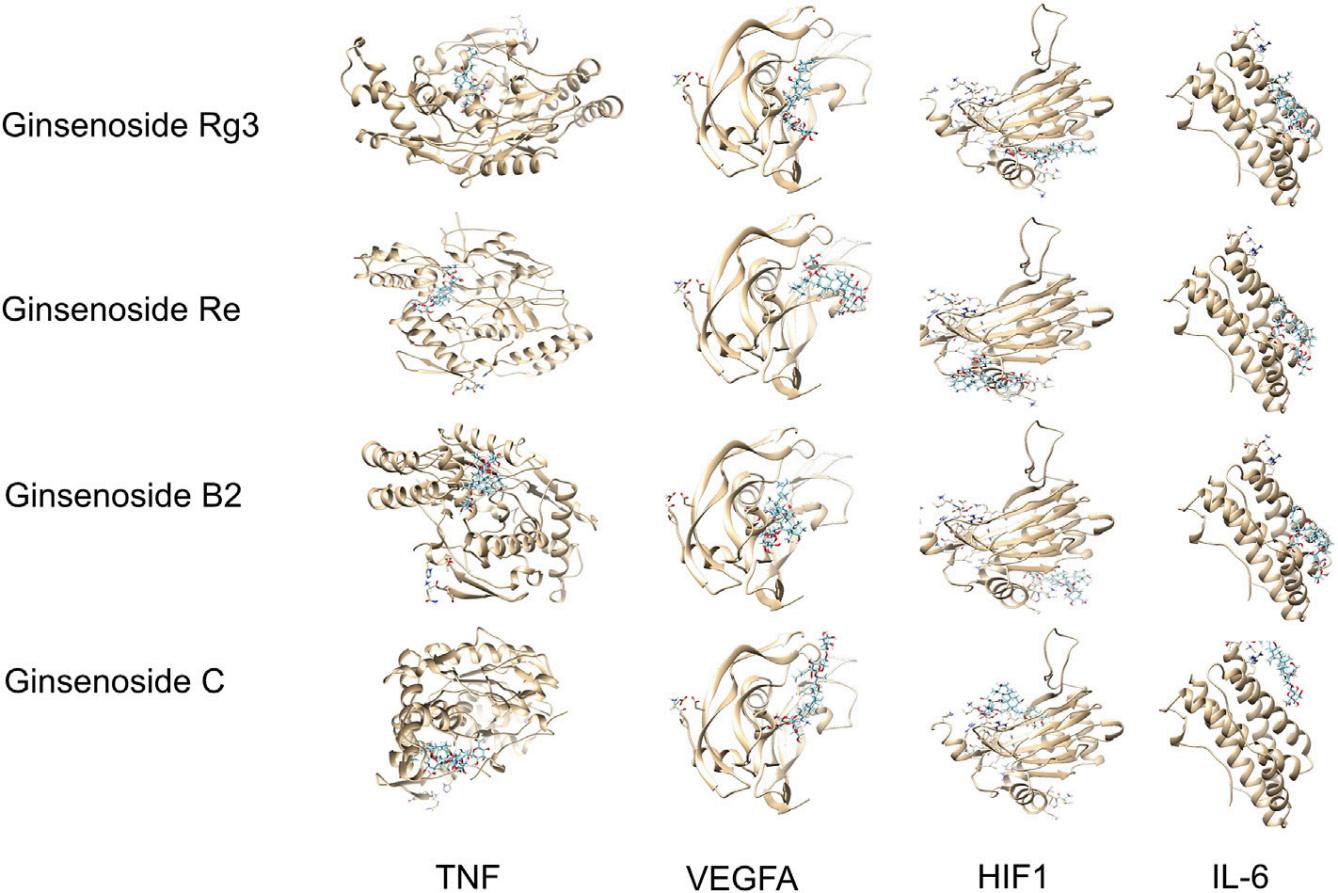
Molecular docking results of selected components and targets.

The active ingredients of ginsenosides Rg3, Re, B2, and C and the key genes of TP53, STAT3, NOS2, and TNF were selected. Molecular docking results showed that different compounds had a good binding ability to key genes, and the results from the network pharmacology screening were also directly verified by molecular docking. Part of the docking process is shown in Figure 3 and Table 3.

**TABLE 3:** Docking scores of selected components and targets.

Ingredient	Target	Docking score	Ingredient	Target	Docking score
Ginsenoside Rg3	VEGFA	−7.77	Ginsenoside Rg3	HIF1	−9.60
Ginsenoside Re	VEGFA	−9.42	Ginsenoside Re	HIF1	−8.10
Ginsenoside B2	VEGFA	−7.59	Ginsenoside B2	HIF1	−9.24
Ginsenoside C	VEGFA	−7.44	Ginsenoside C	HIF1	−9.28
Ginsenoside Rg3	IL-6	−7.50	Ginsenoside Rg3	TNF	−8.12
Ginsenoside Re	IL-6	−9.41	Ginsenoside Re	TNF	−9.24
Ginsenoside B2	IL-6	−8.69	Ginsenoside B2	TNF	−9.03
Ginsenoside C	IL-6	−10.06	Ginsenoside C	TNF	−7.77

### 3.3 Experiment verification

#### 3.3.1 Mountain-cultivated ginseng and garden ginseng promote the growth of subintestinal vessel plexus in zebrafish

**FIGURE 4:**
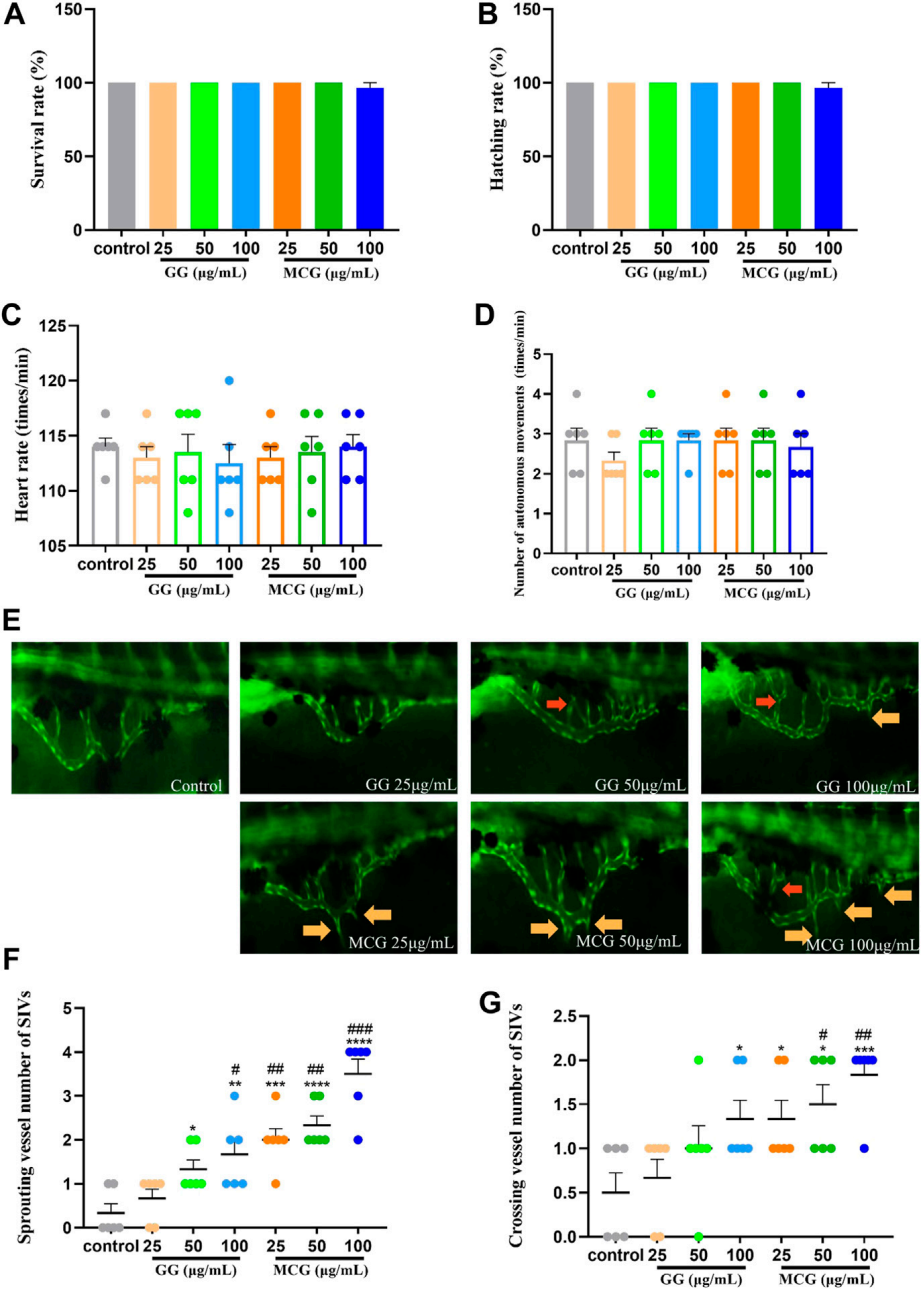
Effect of MCG and GG on subintestinal vessel plexus (SIV) growth in zebrafish. Healthy embryos that were developed 24 h post-fertilization (hpf) were removed and treated with various concentrations of MCG and GG (25, 50, 100 μg/ml) for 48 h. **(A,B)** Survival rate and hatching rate of 72 hpf zebrafish embryos. **(C,D)** Heart rate and number of autonomous movements of 72 hpf zebrafish embryos. **(E)** SIV growth after MCG and GG treatment in zebrafish. Red arrows indicate the crossing vessels, and yellow arrows indicate the sprouting vessels of SIVs. **(F,G)** Sprouting and crossing vessel numbers of SIVs were calculated in each embryo, and data are represented as the mean ± standard error of the mean. *n* = 3. *p < 0.05, **p < 0.01, ***p < 0.001, ****p < 0.0001 versus the control group. ^#^ *p* < 0.05, ^##^ *p* < 0.01, ^###^ *p* < 0.001 compared with the GG (25 μg/ml)-treated group.

The tolerance of zebrafish embryos to MCG and GG is depicted in Supplementary Figure S1. Regression analysis showed that the lowest lethal concentration of MCG and GG was 136.25 μg/ml. To ensure the normal survival of zebrafish embryos during the experiment, different concentrations of MCG and GG (25, 50, and 100 μg/ml) were selected for subsequent angiogenesis promotion experiments. In a healthy zebrafish model, the SIV region is a smooth arched structure similar to a basket net. Compared with the zebrafish in the control group, no significant differences in the survival rate, hatching rate, heart rate, and autonomous movements were observed in the zebrafish in the MCG and GG treated groups (Figures 4A–D); however, the sprouting and crossing of SIVs occurred 48 h after MCG and GG treatment. The amount of sprouted and crossed vessels was positively correlated with the MCG concentration used for treatment (Figures 4E–G). Both MCG and GG have a promotive effect on the growth of SIVs in zebrafish; however, compared with GG, MSG has a stronger promotive effect.

#### 3.3.2 Mountain-cultivated ginseng and garden ginseng promote the growth of intersegmental vessels in zebrafish

**FIGURE 5:**
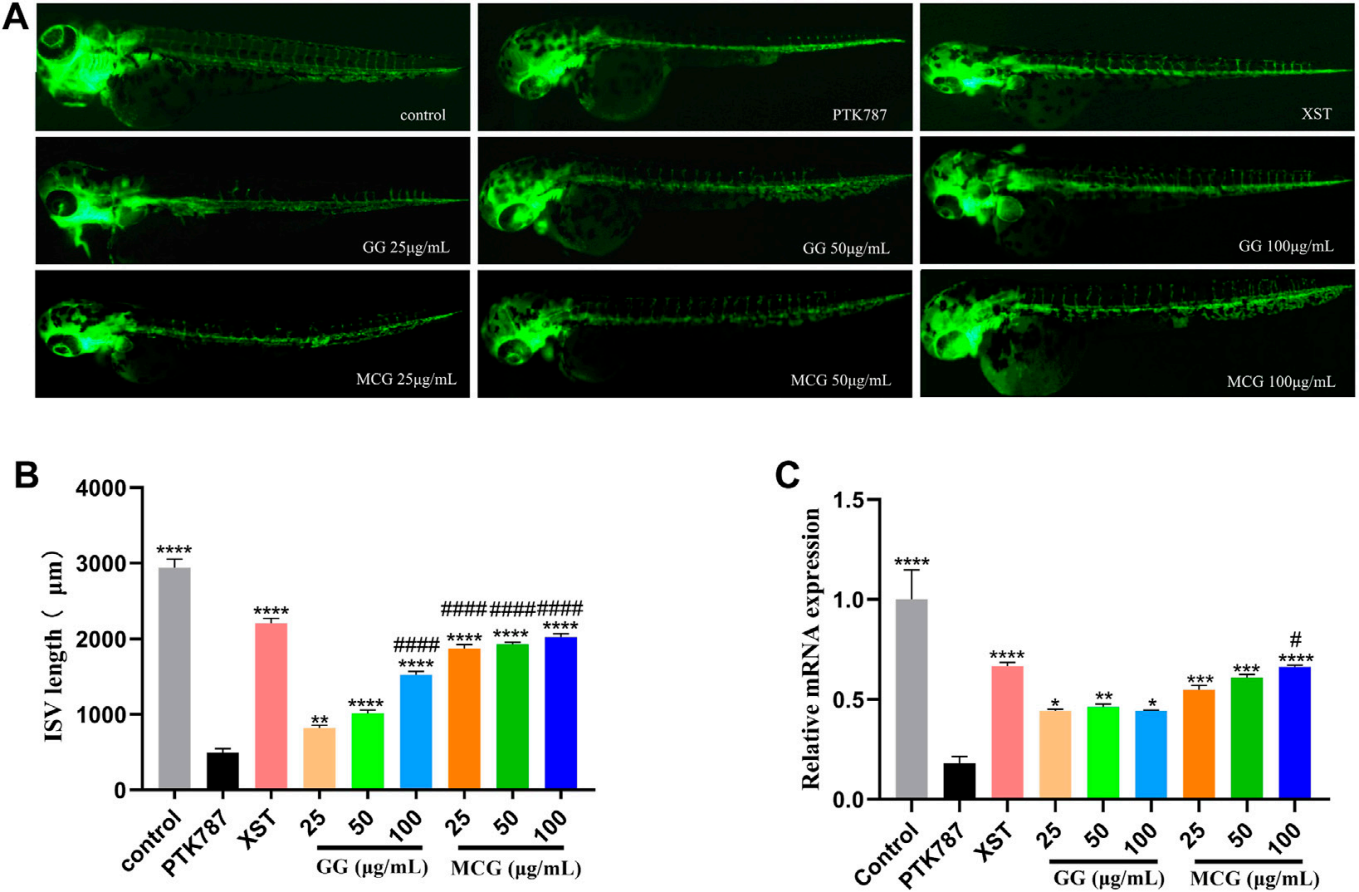
Effect of MCG and GG on intersegmental vessel (ISV) growth in zebrafish. The embryos at 21 hpf were pretreated with 0.2 μg/ml PTK787 (VEGF receptor inhibitor) for 3 h and then treated with various concentrations of MCG and GG (25, 50, 100 μg/ml) for 48 h. **(A)** ISV growth after MCG and GG treatment in zebrafish. **(B)** ISV length was calculated in zebrafish embryos. **(C)** The mRNA expression of VEGFR2 was analyzed using RT-PCR. Data are represented as the mean ± standard error of the mean. *n* = 3. *p < 0.05, **p < 0.01, ***p < 0.001, ****p < 0.0001 versus the PTK787 group; ^#^ *p* < 0.05, ^####^ *p* < 0.0001 compared with the GG (25 μg/ml)-treated group.

The embryos were pretreated with PTK787 to establish the faulty angiogenesis model. PTK787 is a type of VEGF receptor inhibitor. The ISVs in zebrafish in the control group grew well without injury and were present in a uniform, longitudinal equidistant manner; however, the ISVs in the zebrafish in the PTK787 group eventually disappeared and were damaged (Figure 5A). MCG and GG treatment significantly reduced the inhibition of PTK787 on the ISVs in a dose-dependent pattern. Compared with GG, MCG had a greater therapeutic effect on the inhibition of PTK787 (Figure 5B). Meanwhile, the negative and positive correlations of VEGFR2 expression with PTK787 and MCG and GG treatment, respectively, were also noted (Figure 5C).

#### 3.3.3 Effect of the hypoxia-inducible factor-1α/vascular endothelial growth factor signaling pathway on the proangiogenesis effect of mountain-cultivated ginseng and garden ginseng

**FIGURE 6:**
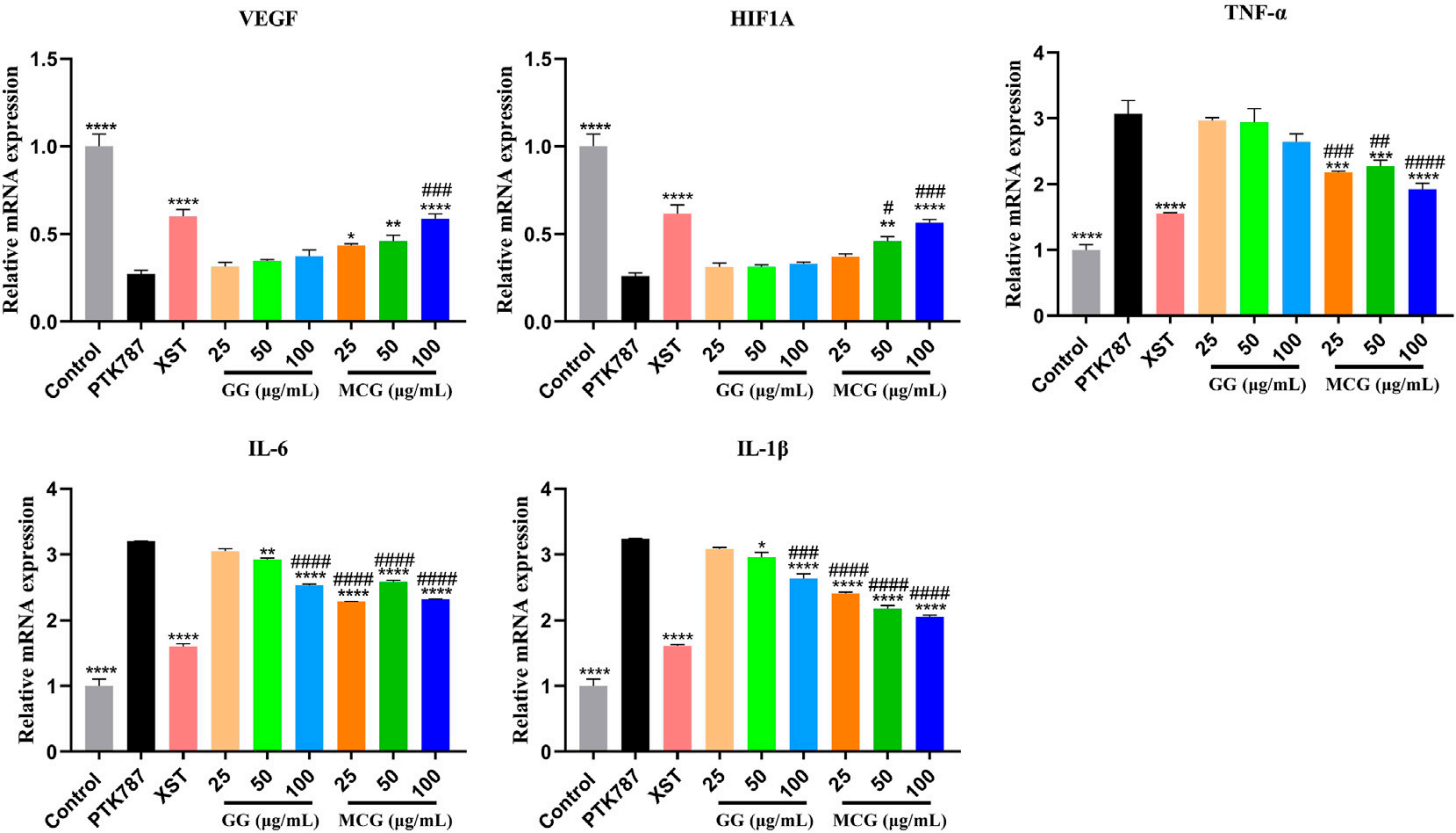
Effects of MCG and GG on the targets related to angiogenesis by RT-PCR. Relative mRNA expression levels of VEGF, HIF1A, TNF-α, IL-6, and IL-1β. Data are represented as the mean ± standard error of the mean. *n* = 3. *p < 0.05, **p < 0.01, ***p < 0.001, ****p < 0.0001 versus the PTK787 group; ^##^ *p* < 0.01, ^###^ *p* < 0.001, ^####^ *p* < 0.0001 compared with the GG (25 μg/ml)-treated group.

Using network pharmacological analysis, the hypotheses of these potential targets and signaling pathways were confirmed and the molecular mechanism of the proangiogenesis effect of MCG and GG was predicted, which suggested the strong association between the proangiogenesis effect of MCG and GG and the PI3K/AKT and HIF-1α/VEGF signaling pathways and inflammatory factors. The RT-PCR results revealed that compared with the control group, PTK787 treatment significantly decreased the HIF-1α and VEGF levels and increased the TNF-α, IL-1β, and IL-6 levels, whereas compared with the PTK787-treated group, MCG and GG treatment significantly increased the HIF-1α and VEGF mRNA expression and decreased the TNF-α, IL-1β, and IL-6 expression (Figure 6). In addition, MCG exhibited a stronger effect than GG.

## 4 Discussion

Multiple studies have shown that ginseng has several pharmacological benefits, including the prevention and treatment of CVD, as well as a wide range of clinical applications. The secondary metabolites of MCG and GG differ due to the differences in the growth environment, resulting in differences in the pharmacological actions of the two types of ginseng. Therefore, it is of great clinical significance to explore the differences in the pharmacological effects of MCG and GG.

In this study, the pharmacological effects of MCG and GG on vascular growth, which can increase angiogenesis, help minimize the extent of myocardial infarction, and protect the ischemic myocardium, were studied using zebrafish as a model animal. Both types of ginseng had no effect on the survival rate and heart rate of zebrafish embryos, indicating low toxicity and cardiotoxicity. MCG and GG improved the number of sprouting and crossing vessels in zebrafish SIVs, with MCG having a much greater effect than GG. Likewise, both types of ginseng could protect damaged ISVs, with MCG having a substantially greater effect than GG and MCG having a vascular growth rate of 73% at 100 g/ml. Taken together, our present study showed that MCG and GG have the bioactivity to promote angiogenesis in a pathophysiological state, and MCG has significant efficacy in the recovery of blood vessel loss.

Several studies on the identification of ginseng and the determination of the ginsenoside content have recently been published; however, there have been few investigations on the differences in medicinal components between MCG and GG. Ginsenoside Rg3, ginsenoside Re, ginsenoside B2, ginsenoside C, L-phenylalanine, and ginsenoside M7cd expression levels were shown to be higher in MCG than in GG. Meanwhile, 1-palmitoyl lysophosphatidic acid, ginsenoside F5, citric acid, N2-acetyl-L-ornithine, and notoginsenoside Fa expression levels were lower in MCG than in GG, with 1-palmitoyl lysophosphatidic acid being the most significantly differentiated secondary metabolite, which was important for the identification of 1-palmitoyl lysophosphatidic acid as a quality marker of ginseng. The protective effects of ginsenoside Rg3 (Lee et al., 2016; Jeong DIrfan et al., 2017; Kwon, 2018) on the cardiovascular system, including the inhibition of platelet aggregation, antithrombosis, and coronary artery dilation, have been observed. Ginsenoside Re (Chen et al., 2016; Wang et al., 2018; Wang et al., 2019; Yu et al., 2020) has been shown to promote angiogenesis and related cardiac electrophysiological activities. Ginsenoside C (Xue et al., 2020) can protect against ischemia-reperfusion injury (Huang et al., 2014; Miao et al., 2020; Sun et al., 2020). Gensenosides B2, C, F5, and M7cd are rarely studied with regards to their pharmacological effects. As a result, future research on their chemical structure, structural similarity to other components, and pharmacological effects will be needed. Furthermore, the transformation of uncommon ginsenosides can be employed as a pivotal point in metabolite differentiation studies.

MCG has been growing in the wild for over 15 years; its shape, color, fragrance, taste, and other characteristics are outstanding, and it has substantial medical significance in clinical applications. Ginseng has been used in the treatment of CVDs such as heart failure and coronary atherosclerotic heart disease in clinical trials. Some medicine has been widely used in the treatment of CVD, such as Rinchen Decoction and Shenfu Decoction; however, the differences in the pharmacological effects of MCG and GG in the treatment of CVD remain largely undetermined. VEGF is an important signal protein involved in angiogenesis and endothelial cell growth, thereby promoting the function of angiogenesis. VEGF can specifically bind to its corresponding receptor (VEGFR) and stimulate the proliferation of vascular endothelial cells, promoting the formation of collateral vessels. HIF-1 (Li et al., 2020; Liu et al., 2020; Zhang et al., 2021) is a key transcriptional mediator of the hypoxic conditioned response. The HIF-1 pathway is a major regulator of angiogenesis, which is synergistic with other proangiogenic factors. In the zebrafish vascular injury model, both MCG and GG can upregulate the VEGF and HIF-1α expression, and MCG has a stronger pharmacological effect than GG. Taken together, MCG and GG have substantial differences in the repair of vascular injury, and metabolomic analysis could also be a potential tool for studying CVD drugs. In addition, these results provide data references for the clinical selection of MGG and GG. In particular, our results prove that MCG has potential for clinical application.

## 5 Conclusion

In conclusion, we used plant metabolomics and network pharmacology to determine the angiogenic activity of MCG and GG. Based on the results of the zebrafish experiments, both MCG and GG can reduce inflammation and promote angiogenesis via the HIF-1α/VEGF pathway. Our findings showed that MCG has a higher metabolite content than GG and that these metabolites enhanced angiogenesis and had stronger pharmacological activity in CVD than those of GG. The accuracy of the predicted findings will be improved using our proposed method, which used experimentally determined constituents and their matching targets. The current work was the first to integrate metabolomics with zebrafish experiments to investigate the effects of MCG and GG on CVD, providing some theoretical support for clinical treatment.

## Data Availability

The original contributions presented in the study are included in the article/Supplementary Material, further inquiries can be directed to the corresponding author.
