# Supplementary figures and images for: Investigation of the pharmacological effect and mechanism of mountain-cultivated ginseng and garden ginseng in cardiovascular diseases based on network pharmacology and zebrafish experiments
Source: Front Pharmacol. 2022 Sep 1;13:920979. doi: 10.3389/fphar.2022.920979 (PMC9474728; doi:10.3389/fphar.2022.920979)

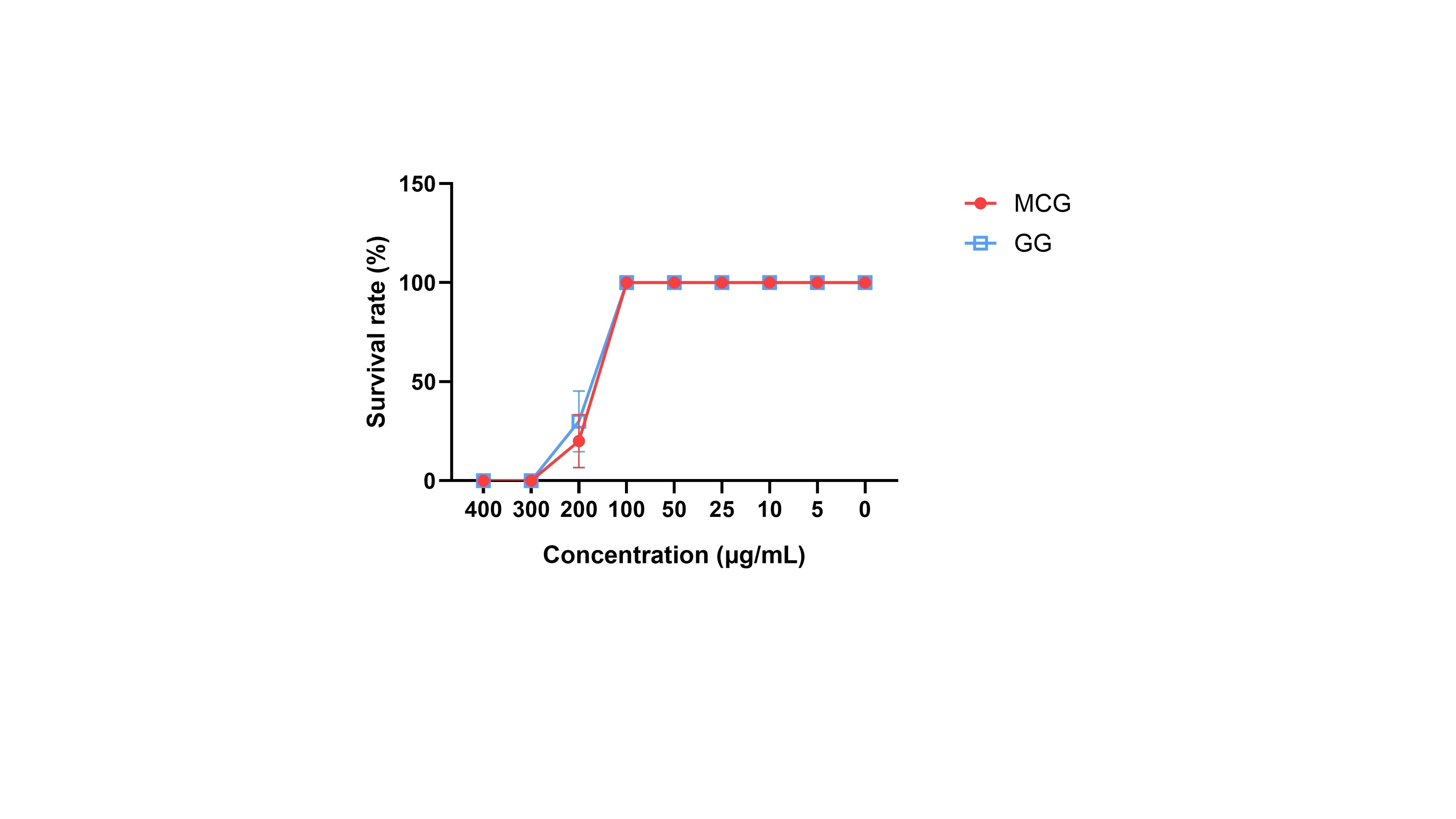

Supplement: Supplementary file 4 [file Image1.JPEG]
