# Supplementary material for: Investigation of the pharmacological effect and mechanism of mountain-cultivated ginseng and garden ginseng in cardiovascular diseases based on network pharmacology and zebrafish experiments
Source: Front Pharmacol. 2022 Sep 1;13:920979. doi: 10.3389/fphar.2022.920979 (PMC9474728; doi:10.3389/fphar.2022.920979)
Supplement: Supplementary file 5 [file DataSheet1.docx]

**Mechanisms exploration of Mountain-and Garden-Cultivated Ginseng on cardiovascular disease based on UPLC-Q-TOF/MS and Zebrafish model**

***Supplemental Information***

**Effects of MCG and CG on the growth of zebrafish**

Zebrafish embryos developed to 24 hpf were randomly allocated into 24-well plates, 10 in each well, and eight different concentrations (5, 10, 25, 50, 100, 200, 300 and 400 μg/mL) of MCG and CG were investigated respectively. The tolerance of zebrafish embryos to MCG and CG were shown in Figure S1. All zebrafish embryos died when the concentration was 400 and 300 μg/mL;

the death rate of MCG group was 80%, and that of CG group was 70% in 200 μg/mL group; zebrafish embryos did not die and no obvious toxic phenotype was found when the concentration was below 100 μg/mL. Regression analysis showed that the lowest lethal concentration of MCG and CG was 136.25 μg/mL. In order to ensure the normal survival of zebrafish embryos during the experiment, MCG and CG was selected to give 25, 50 and 100 μg/mL of drug for subsequent angiogenesis promotion experiments.

**Supplemental Figure Legends**

**Fig S1** Effects of MCG and CG on the growth of zebrafish.
